# PPAR*γ* and Apoptosis in Cancer

**DOI:** 10.1155/2008/704165

**Published:** 2008-07-02

**Authors:** Heath A. Elrod, Shi-Yong Sun

**Affiliations:** Department of Hematology and Medical Oncology, Winship Cancer Institute, Emory University School of Medicine, Atlanta, GA 30322, USA

## Abstract

Peroxisome proliferator-activated receptors (PPARs) are ligand binding transcription factors which function in many physiological roles including lipid metabolism, cell growth, differentiation, and apoptosis. PPARs and their ligands have been shown to play a role in cancer. In particular, PPAR*γ* ligands including endogenous prostaglandins and the synthetic thiazolidinediones (TZDs) can induce apoptosis of cancer cells with antitumor activity. Thus, PPAR*γ* ligands have a potential in both chemoprevention and therapy of several types of cancer either as single agents or in combination with other antitumor agents. Accordingly, the involvement of PPAR*γ* and its ligands in regulation of apoptosis of cancer cells have been extensively studied. Depending on cell types or ligands, induction of apoptosis in cancer cells by PPAR*γ* ligands can be either PPAR*γ*-dependent or -independent. Through increasing our understanding of the mechanisms of PPAR*γ* ligand-induced apoptosis, we can develop better strategies which may include combining other antitumor agents for PPAR*γ*-targeted cancer chemoprevention and therapy. This review will highlight recent research advances on PPAR*γ* and apoptosis in cancer.

## 1. INTRODUCTION

Peroxisome proliferator-activated receptors (PPARs) are ligand binding transcription factors belonging to the nuclear receptor superfamily which includes receptors for steroids, thyroid hormone, and retinoids [1, 2]. PPARs function in a variety of roles including regulation of lipid metabolism, immune function, cell growth, differentiation, and apoptosis [2]. PPARs are involved in several diseases including obesity, diabetes, cardiovascular disease, and cancer [3]. Three different subtypes of PPARs have been identified, PPAR*α*, PPAR*β*/*δ*, and PPAR*γ*, each encoded by separate genes. The three isoforms share functions as well as have distinct activities [2].

PPARs function by regulating gene transcription via binding to DNA sequences known as peroxisome proliferator response elements (PPREs) located in the promoter regions of target genes. PPREs are direct repeats of the consensus sequence with a spacing of one nucleotide (AGGTCA N AGGTCA) [4]. PPARs bind to PPREs as heterodimers with retinoid X receptors (RXR). The heterodimer PPAR/RXR can bind other transcriptional coactivators or corepressors to influence gene transcription [1]. Ligand binding to PPARs induces conformational changes that release corepressors from the heterodimer and recruit coactivators to allow for target gene transcription [5].

Synthetic and endogenous PPAR ligands have been used to elucidate the role of PPARs. Specifically, thiazolidinediones (TZDs) including pioglitazone, ciglitazone, troglitazone, and rosiglitazone are synthetic PPAR*γ* ligands which are insulin-sensitizing agents developed to treat diabetes mellitus [2]. The naturally occurring prostaglandin, 15-deoxy-Δ^12,14^-prostaglandin J_2_(15d-PGJ_2_), is generally considered to be an endogenous PPAR*γ* ligand [6, 7]. The promiscuous nature of PPARs may lead to the binding of multiple ligands resulting in the activation of many cellular pathways. These ligands have been extensively studied and shown to exert antineoplastic properties including induction of apoptosis.

**Figure 1:**
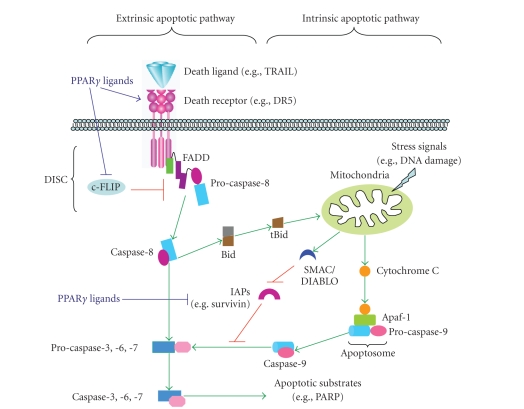
*Schema for basic apoptotic signaling pathways and possible mechanisms underlying PPAR*γ* ligand-induced apoptosis*. Ligation of death ligands (e.g., TRAIL) with their receptors (e.g., DR5) results in formation of the death-inducing signaling complex (DISC), in which pro-caspase-8 will be recruited through the death adaptor protein FADD and cleaved to generate activated caspase-8. This process is inhibited by c-FLIP. Certain stress signals (e.g., DNA damage) can target mitochondria and induce cytochrome C release from the mitochondria into the cytosol leading to caspase-9 activation by forming an apoptosome via binding to Apaf-1. Both caspase-8 and caspase-9 activate downstream procaspase-3, -6, and -7, leading to cleavages of their target death proteins such as PARP. In addition, truncated Bid (tBid), activated by caspase-8 via cleavage, facilitates insertion of Bax into the mitochondrial membrane leading to cytochrome C release. Therefore, tBid may serve as a link between the extrinsic and intrinsic apoptotic pathways. Inhibitors of apoptosis proteins (IAPs) such as survivin can bind to activated caspase-9 and prevent its action on effector caspases, whereas SMAC/DIABLO binds to IAPs, leaving caspase-9 free to activate the effector caspases. PPAR*γ* ligands may induce apoptosis through induction of DR5 and/or downregulation of c-FLIP and/or survivin.

Apoptosis or programed cell death is a highly regulated process critical for normal development and tissue homeostasis. Aberrant regulation of apoptosis can lead to cancer. Apoptosis is induced from signals inside or outside the cell including radiation, viral infection, growth factors, and hormones [23]. Apoptosis involves signature morphological changes induced by caspases, which are activated upon induction of apoptotic signaling and cleave downstream molecules to facilitate the apoptotic cascade [24]. The induction of apoptosis can occur through two pathways: the intrinsic apoptotic pathway which involves signaling through the mitochondria and the extrinsic apoptotic pathway which is initiated through activation of cell surface death receptors [25]. Apoptotic signaling through the intrinsic pathway primarily involves activation of the proapoptotic Bcl-2 family members Bax and Bak, which facilitate release of cytochome C from the mitochondria and subsequent caspase-9 cleavage or activation. The activated caspase-9 will finally cleave or activate the downstream effector caspases such as caspase-3 and -7, leading to apoptosis. This pathway is negatively regulated by several antiapoptotic Bcl-2 family members such as Bcl-2 and Bcl-X_L_ [26]. Apoptotic signaling through the extrinsic pathway is initiated by ligand binding to death receptors or by induction of trimerization of the receptors [27]. The death receptors belong to the tumor necrosis factor (TNF) receptor superfamily, which includes Fas, TNFR1, DR3, DR4 (TRAIL-R1), DR5 (TRAIL-R2), and DR6. Upon ligand binding and trimerization of death receptors, the intracellular death domain of the death receptors recruits adapter proteins such as Fas-associated death domain (FADD), forming a death-inducing signaling complex (DISC) which helps recruit procaspase-8 to the DISC. Caspase-8 is then activated, leading to activation of the downstream effector caspases such as caspase-3 and -7. The effector caspases can also be activated by death receptors indirectly through caspase-8-mediated cleavage of Bid, which facilitates Bax activation and subsequent release of cytochome C from the mitochondria. Thus, the Bid cleavage links the two apoptotic pathways [28]. Cellular FLICE inhibitory protein (c-FLIP), an inactive homolog of caspase-8, primarily functions as an inhibitor of the extrinsic apoptotic pathway by preventing caspase-8 activation, whereas inhibitors of apoptosis protein (IAPs) such as survivin mainly suppress the intrinsic apoptotic pathway by inhibiting caspase-9 as well as caspase-3 activation (Figure 1).

PPARs, particularly PPAR*γ*, and their ligands play a role in regulation of both apoptotic pathways. Thus, this review will specifically focus on the role of PPAR*γ* and its ligands in regulation of tumor cell apoptosis. Some of the underlying mechanisms resulting in apoptosis of tumor cells in PPAR*γ*-dependent and -independent manners will be highlighted.

## 2. PPAR*γ* AGONISTS INDUCE APOPTOSIS OF CANCER CELLS

PPAR*γ* agonists (e.g., TZDs) have been shown to induce apoptosis in a variety of cancer cells including lymphoma, multiple myeloma, bladder, gastric, esophageal, pancreatic, hepatoma, colon, breast, brain, and lung cancer cells [8, 12, 29–39]. However, many of the underlying mechanisms of the apoptotic properties of TZDs remain unknown. In general, this induction of apoptosis is PPAR*γ*-dependent and/or -independent depending on cell types or ligands (Table 1).

### 2.1. PPAR*γ*-dependent apoptosis

In thyroid cancer cell lines, it has been shown that the expression of PPAR*γ* correlates with the sensitivity of troglitazone and 15d-PGJ_2_ to cell death. Thyroid cancer cells that did not express PPAR*γ* showed no growth inhibition after treatment with troglitazone and 15d-PGJ_2_ compared with thyroid cancer cells that did express PPAR*γ* and were sensitive to growth inhibition by troglitazone and 15d-PGJ_2_, suggesting PPAR*γ*-dependent growth inhibition. Growth inhibition by troglitazone was due to apoptosis as was seen by DNA laddering [9]. Another study in thyroid cancer cell lines also implicates PPAR*γ* as an important target. In this study, ciglitazone was effective in reducing the growth of thyroid cancer cells that expressed PPAR*γ*, but had no effect in reducing growth in a thyroid cancer cell line that do not express PPAR*γ* [10]. After introduction of wild-type PPAR*γ* into the PPAR*γ*-deficient cells, these cells became responsive to ciglitazone. Moreover, overexpression of PPAR*γ* in thyroid cancer cells significantly increased apoptosis compared to cells transfected with empty vector or with a vector carrying a mutated nonfunctional PPAR*γ* cDNA [10]. Collectively, it appears that the presence of PPAR*γ* at least partly contributes to the induction of apoptosis by PPAR*γ* ligands in thyroid cancer. The recent findings from a transgenic mouse study [11] may provide an explanation for why thyroid cancer is susceptible to treatment with PPAR*γ* agonists. Mice harboring a knockin dominant negative mutant thyroid hormone receptor *β* (TR*β*PV/PV mouse) spontaneously develop follicular thyroid carcinoma similar to human thyroid cancer. Using the offspring from the cross of TR*β*PV/+ and PPAR*γ*+/− mice, Kato et al. [11] found that thyroid carcinogenesis progressed significantly faster in TR*β*PV/PV mice with PPAR*γ* insufficiency from increased cell proliferation and reduced apoptosis. Reduced PPAR*γ* protein activated the NF-*κ*B signaling pathway, resulting in the activation of cyclin D1 and repression of critical genes involved in apoptosis. Treatment of TR*β*PV/PV mice with a PPAR*γ* agonist, rosiglitazone, delayed the progression of thyroid carcinogenesis by decreasing cell proliferation and activating apoptosis. These results suggest that PPAR*γ* is a critical modifier in thyroid carcinogenesis.

Other molecular mediators of apoptosis have been examined in PPAR*γ*-dependent models. In thyroid cancer cells, troglitazone increased c-Myc expression without changing the expression of Bcl-2 or Bax [9]. In contrast, in colon cancer cells, both troglitazone and 15d-PGJ_2_ have been shown to downregulate c-Myc expression [12]. Thus, whether c-Myc is involved in mediating PPAR*γ* agonist-induced apoptosis needs further investigation.

In colon and lung cancer cells, troglitazone was reported to increase the expression of growth arrest and DNA-damage inducible 153 (GADD153) [12, 13], a key apoptosis-regulated gene particularly involved in endoplasmic reticulum (ER) stress-induced apoptosis [40]. Further analysis revealed that troglitazone did not stimulate GADD153 mRNA levels in undifferentiated 3T3-L1 cells lacking PPAR*γ* expression, whereas its induction was clearly observed in differentiated adipocytes expressing PPAR*γ*, suggesting the importance of PPAR*γ* in troglitazone-induced GADD153 expression. In lung cancer cells, inhibition of GADD153 gene expression by an antisense phosphorothionate oligonucleotide attenuated the troglitazone-induced growth inhibition [13]. These findings collectively suggest that GADD153 might be a candidate factor implicated in TZD-induced growth inhibition and apoptosis.

Several studies have demonstrated the importance of ERK and its regulated genes in PPAR*γ* agonist-induced apoptosis [14–16, 41]. In human lung cancer cells, troglitazone induced apoptosis as well as PPAR*γ* and ERK1/2 accumulation in the nucleus. Both PPAR*γ* siRNA and U0126, a specific inhibitor of ERK1/2, blocked these effects of troglitazone, suggesting that troglitazone-induced apoptosis is PPAR*γ*- and ERK1/2-dependent. Moreover, inhibition of ERK1/2 by U0126 also significantly decreased the levels of PPAR*γ*, suggesting a positive crosstalk between PPAR*γ* and ERK1/2 or an autoregulatory feedback mechanism to amplify the effect of ERK1/2 on cell growth arrest and apoptosis [16].

Proline oxidase (POX) is a redox enzyme localized in the mitochondrial inner membrane and functions as a p53-induced gene that can mediate apoptosis through generation of reactive oxygen species (ROS) [17]. A recent study in colon cancer cells showed that troglitazone enhanced the binding of PPAR*γ* to PPRE in the POX promoter, activated the POX promoter, and increased endogenous POX expression. Blocking of PPAR*γ* activation either by the antagonist GW9662 or deletion of the PPAR-responsive element in the POX promoter only partially decreased the POX promoter activation in response to troglitazone, suggesting also the involvement of PPAR*γ*-independent mechanisms. Further, troglitazone induced p53 protein expression in HCT116 cells, which may be the possible mechanism for PPAR*γ*-independent POX activation, since POX has been shown to be a downstream mediator in p53-induced apoptosis. In HCT15 cells, with both mutant p53 and mutant PPAR*γ*, troglitazone did not activate POX, whereas it did in HT29 cells, with a mutant p53 and wild type PPAR*γ*, indicating that both PPAR*γ*-dependent and -independent mechanisms are involved in the troglitazone-induced POX expression [17]. Thus, this study suggests that troglitazone-induced apoptosis involves targeting POX gene expression for generation of ROS.

### 2.2. PPAR*γ*-independent apoptosis

To help discern the PPAR*γ*-dependent and -independent properties of TZDs, TZD derivatives lacking PPAR*γ* activity were developed. These derivatives have a double bond adjoining the terminal thiazolidine-2,4-dione ring which abolishes ligand binding to PPAR*γ* [42]. Shiau et al. [18] showed that the pioglitazone, troglitazone, and ciglitazone derivatives (Δ2-PG, Δ2-TG, Δ2-CG) were unable to activate PPAR*γ* compared to pioglitazone, troglitazone, and ciglitazone which showed significant activation of PPAR*γ*. When troglitazone and Δ2-TG were tested for growth inhibition in two prostate cancer cell lines: one cell line expressing high levels of PPAR*γ* (PC-3) and one deficient of PPAR*γ* expression (LNCaP), the LNCaP cells were more sensitive to troglitazone compared to PC-3 cells despite being deficient in PPAR*γ*. As well, Δ2-TG which cannot activate PPAR*γ* was more effective than troglitazone in suppressing growth in both PC-3 and LNCaP cells. Both troglitazone and Δ2-TG induced cytochome C release and DNA fragmentation in these cells, attributing the growth inhibition to apoptosis. These results suggest that TZDs can induce apoptosis independent of PPAR*γ* activation. The induction of apoptosis in this study appears to be partly due to the inhibition of the antiapoptotic function of Bcl-2 and Bcl-X_*L*_. It is thought that Bcl-2 and Bcl-xL sequester proapoptotic molecules such as Bax and Bak through heterodimerization through BH3 domain binding which inhibits the proapoptotic function of Bax and Bak [43, 44]. Both troglitazone and Δ2-TG reduced the association of Bak with Bcl-2 and Bcl-X_*L*_ causing the cells to undergo apoptosis as shown by cytochrome C release and caspase-9 activation. Moreover, Bcl-X_*L*_ overexpression protected LNCaP cells from troglitazone- and Δ2-TG-induced apoptosis [18]. Collectively, these results show that PPAR*γ* ligands trigger apoptosis independent of PPAR*γ* and primarily target activation of the intrinsic apoptotic pathway, at least in the tested prostate cancer cells.

It was shown that 15d-PGJ_2_, but not rosiglitazone and ciglitazone, induced apoptosis in oral squamous cell carcinoma cells, suggesting that 15d-PGJ_2_ is acting through pathways other than activation of PPAR*γ*. In this study, the apoptotic effect of 15d-PGJ_2_ was associated with down regulation of the oncogene Stat3 which was not seen with rosiglitazone or ciglitazone [19]. Similarly, in bladder and prostate cancer cells, 15d-PGJ_2_ and troglitazone inhibited cell growth but rosiglitazone and pioglitazone had no effect on growth inhibition. 15d-PGJ_2_ inhibited cell growth by induction of apoptosis, while troglitazone induced cell cycle arrest [20]. Thus, the induction of apoptosis can also be selective for certain PPAR*γ* ligands.

Other mediators of apoptosis in PPAR*γ* ligand-induced cell death include the early growth response-1 (EGR-1) transcription factor. EGR-1 has been linked to apoptosis and shown to be activated by ERK. In colon cancer cells, EGR-1 was induced dramatically by troglitazone but not by other PPAR*γ* ligands [14]. Inhibition of ERK phosphorylation abolished EGR-1 induction by troglitazone, suggesting an ERK-dependent induction of EGR-1. Given that troglitazone-induced apoptosis is accompanied by the biosynthesis of EGR-1, these results suggest that PPAR*γ*-independent EGR-1 induction is a unique property of troglitazone compared with other PPAR*γ* ligands and may play an important role in troglitazone-induced apoptosis [14]. One of the EGR-1-regulated genes is proapoptotic nonsteroidal anti-inflammatory drug (NSAID)-activated gene (NAG-1) [15]. A recent study has demonstrated that the novel TZD derivative MCC-555 exerts a PPAR*γ*-independent upregulation of NAG-1. Moreover, NAG-1 induction contributes to MCC-555-induced apoptosis as downregulation of NAG-1 by siRNA suppressed MCC-555-induced apoptosis [41]. As well, NAG-1 induction was also observed in colon cancer cells treated with troglitazone or 15d-PGJ_2_ [15]. Importantly, both agents induce NAG-1 expression through an EGR-1-dependent mechanism. However, troglitazone, but not 15d-PGJ_2_, increases EGR-1 binding to the EGR-1 binding site located within region -73 to -51 of the NAG-1 promoter; this effect has an important role in the transactivation of TGZ-induced NAG-1 expression. The effect of 15d-PGJ_2_ is probably PPAR*γ*-dependent because a PPAR*γ* antagonist inhibited the 15d-PGJ_2_-induced expression of NAG-1, whereas TGZ-induced NAG-1 expression was not inhibited by the PPAR*γ* antagonist [15].

Multiple myeloma and Burkitt lymphoma cells express constitutively active NF-*κ*B. 15d-PGJ_2_ was reported to suppress constitutive NF-*κ*B activity and potently induce apoptosis in both types of B-cell malignancies. NF-*κ*B inhibition is accompanied by rapid downregulation of NF-*κ*B-dependent antiapoptotic gene products, including cellular inhibitor-of-apoptosis protein 1 (cIAP-1), cIAP-2, X-chromosome-linked inhibitor-of-apoptosis protein (XIAP), and c-FLIP. These effects were mimicked by the proteasome inhibitor MG-132, but not by troglitazone, suggesting that 15d-PGJ_2_-induced apoptosis is independent of PPAR*γ* [21]. Thus, the inhibition of NF-*κ*B may play a major role in the proapoptotic activity of 15d-PGJ_2_ in aggressive B-cell malignancies characterized by aberrant regulation of NF-*κ*B. Another study in MCF7 breast cancer cells has shown that both PPAR*γ* and p53 are involved in rosiglitazone-induced apoptosis. However, the NF-*κ*B sequence in the p53 promoter region is required for rosiglitazone to increase p53 transcription in this study [22].

## 3. PPAR AGONISTS AUGMENT DEATH RECEPTOR-INDUCED APOPTOSIS

Apoptosis induced by death receptors can be initiated through binding of death receptor ligands such as TRAIL or Fas ligand. PPAR*γ* ligands can increase death receptor expression and augment death receptor-induced apoptosis. The linkage between PPAR*γ* and TRAIL/death receptor-induced apoptosis came from the early work showing that the PPAR*γ* ligand pioglitazone enhances TRAIL-induced apoptosis through induction of p21 (WAF1) [45, 46]. Subsequently, there are multiple studies demonstrating that different PPAR*γ* ligands have the ability to enhance TRAIL-induced apoptosis in various types of cancer cells both in vitro and in vivo [34, 47–51]. The majority of the studies using various approaches including PPAR*γ* antagonists, PPAR*γ* siRNA or dominant-negative PPAR*γ* mutants conclude that PPAR*γ* ligands enhance TRAIL/death receptor-induced apoptosis through PPAR*γ*-independent mechanisms [34, 47–49] (Table 2).

Among these studies, Kim et al. [50] first reported their important findings that a variety of natural and synthetic ligands of PPAR*γ* including 15d-PGJ_2_, ciglitazone, troglitazone, and the triterpenoid 2-cyano-3,12-dioxooleana-1,9-dien-28-oic acid (CDDO) selectively reduce the levels of c-FLIP, and hence sensitize tumor but not normal cells to apoptosis induction by TRAIL. Both PPAR*γ* agonists and antagonists displayed these effects, regardless of the levels of PPAR*γ* expression and even in the presence of a PPAR*γ* dominant-negative mutant, indicating a PPAR*γ*-independent mechanism. Importantly, PPAR*γ* agonists induced ubiquitination and proteasome-dependent degradation of c-FLIP, without concomitant reductions in c-FLIP mRNA, thus demonstrating a mechanism by which PPAR*γ* agonists decrease c-FLIP through facilitating ubiquitin/proteasome-dependent c-FLIP degradation.

Our group has shown that PPAR*γ* agonists including ciglitazone, troglitazone, and GW1929 induce the expression of death receptor 5 (DR5) including increasing the cell surface distribution of DR5, reducing the levels of c-FLIP, and enhancing TRAIL-induced apoptosis in human lung cancer cells [34]. When c-FLIP was overexpressed or DR5 was silenced, PPAR*γ* ligands showed diminished ability to enhance TRAIL-induced apoptosis, indicating that both DR5 induction and c-FLIP downregulation are two critical events accounting for enhancement of TRAIL-induced apoptosis by PPAR*γ* ligands. Moreover, we have shown that the modulation of DR5 and c-FLIP expression is independent of PPAR*γ* because the use of a PPAR*γ* antagonist and silencing of PPAR*γ* did not effect the ability of the PPAR*γ* ligands to induce DR5 or downregulate c-FLIP [34].

Consistently, 15d-PGJ_2_ has also been shown to induce DR5 expression and augment TRAIL-induced apoptosis in Jurkat leukemia cells and PC-3 prostate cancer cells. This induction of DR5 by 15d-PGJ_2_ occurs posttranscriptionally through increasing DR5 mRNA stability, which is independent of PPAR*γ* activation because two other PPAR*γ* agonists pioglitazone and rosiglitazone did not upregulate DR5 expression and pretreatment with GW9662, a PPAR*γ* inhibitor did not block the induction of DR5 by 15d-PGJ_2_ [47]. As well, DR5 upregulation contributes to the sensitization of TRAIL-induced apoptosis by 15d-PGJ_2_ since the knockdown of DR5 using DR5 siRNA decreased apoptosis induced by the combination of TRAIL and 15d-PGJ_2_ compared to control cells [47].

In renal cancer cells, rosiglitazone, in addition to downregulating c-FLIP expression, also increases DR5 expression at the mRNA level [49]. The use of a DR5/Fc chimeric protein or DR5 siRNA attenuated rosiglitazone and TRAIL-induced apoptosis, indicating a critical role of DR5 induction in this death process. Interestingly, rosiglitazone induced the generation of ROS, whereas cotreatment with glutathione, which can scavenge ROS, prevented ROS generation, DR5 upregulation, and enhancement of TRAIL-induced apoptosis by rosiglitazone, suggesting that ROS-mediated transcriptional activation of DR5 is important for sensitization of renal cancer cells to TRAIL-induced apoptosis [49].

As well, glioblastoma and neuroblastoma cells can be sensitized to TRAIL-induced apoptosis by troglitazone [51]. In addition to upregulation of DR5 and reduction of c-FLIP, troglitazone downregulated survivin levels in these cells, all of which may explain the synergy observed with troglitazone and TRAIL treatment. Importantly, normal astrocytes did not become sensitive to TRAIL-induced apoptosis when treated with troglitazone, suggesting limited toxicity to normal tissues with this treatment [51]. Downregulation of survivin by PPAR*γ* ligands was also observed in breast cancer cells; this event contributes to enhancement of TRAIL-induced apoptosis because enforced expression of ectopic survivin partially protected cells from troglitazone/TRAIL-induced apoptosis. Different from other studies, this study did not find that troglitazone altered the levels of the key proteins in the death receptor-mediate apoptotic pathway including DR4, DR5, and c-FLIP. Instead, they found that troglitazone decreased cyclin D3 levels through inducing ubiqutin/proteasome-mediated protein degradation. Importantly, cyclin D3 downregulation is associated with troglitazone-induced survivin reduction and enhancement of TRAIL-induced apoptosis in human breast cancer cells as silencing of cyclin D3 reduced the levels of survivin and promoted TRAIL-induced apoptosis [48]. Currently, it is unclear how cyclin D3 regulates survivin expression.

In human glioma cells, it has been shown that troglitazone activates protein-tyrosine phosphatase-1B (PTP-1B), which subsequently reduces phosphotyrosine 705 STAT3 (pY705-STAT3) via a PPAR*γ*-independent pathway [53]. Reduction of pY705-STAT3 in glioma cells caused downregulation of c-FLIP and Bcl-2. When given in combination with TRAIL or caspase-dependent chemotherapeutic agents, such as etoposide and paclitaxel, troglitazone exhibited a synergistic effect by facilitating caspase-8 and -9 activities. Thus, it appears that PTP-1B plays a critical role in the downregulation of activated STAT3, as well as c-FLIP and Bcl-2 [53]. However, it is also not clear how PTP-1B regulates the expression of c-FLIP and Bcl-2.

Although PPAR*γ* agonists downregulate c-FLIP through promoting its degradation, the detailed mechanisms by which PPAR*γ* agonists trigger ubiquitin/proteasome-dependent degradation of c-FLIP are unknown. Moreover, the mechanisms underlying PPAR*γ* agonist-induced upregulation of DR5 expression have not been addressed as well. Nonetheless, the sensitization of TRAIL-induced death by PPAR*γ* agonists may have relevant clinical implications as TRAIL is currently being tested in clinical trials for cancer. Identification of tumors that can overcome TRAIL resistance by treatment with PPAR*γ* agonists will enhance tumor specific targeting by TRAIL and reduce toxicity of normal tissues as TRAIL has been shown to induce death in tumor cells while sparing normal cells.

PPAR*γ* agonists have also been shown to effect Fas-mediated apoptosis. In HT-29 colon cancer cells, there was a synergistic effect on induction of apoptosis when the anti-Fas agonistic antibody, CH11, was combined with 15d-PGJ_2_ or ciglitazone [61]. As well, rosiglitazone sensitized the breast cancer cell line, MDA-MB-231, to the antitumor effects of CH11 as well as TNF-*α* [62]. In uterine leiomyoma cells, ciglitazone downregulated the antiapoptotic protein Bcl-2 and upregulated Bax and Fas while enhancing PARP cleavage and caspase-8 activation, suggesting that ciglitazone induces apoptosis in a Fas- and caspase-dependent mechanism [63].

## 4. PPAR*γ* AGONISTS IN COMBINATION WITH OTHER ANTICANCER AGENTS ENHANCE APOPTOSIS

Combination therapy regimens are effective in the clinical treatment of various cancers. Many studies have shown that combining PPAR*γ* agonists with anticancer agents can further sensitize tumor cells to apoptosis (Table 2). As we continue to elucidate the molecular mechanisms of PPAR*γ* in the regulation of cancer formation and development, combining PPAR*γ* agonists with other targeted anticancer agents may be an effective strategy for chemoprevention and treatment of various cancers.

One such combination involves the 5-lipoxygenase inhibitor MK866. MK866 blocks the 5-lipoxygenase pathway of arachidonic acid metabolism, increases the expression of PPAR*α* and PPAR*γ* in breast cancer cells, and induces apoptosis [64]. As well, in lung cancer cells, MK886 increased PPAR*γ* reporter activity. The combination of MK886 with 15d-PGJ_2_ generated greater growth-inhibitory effects including apoptosis than each single agent alone in A549 lung cancer cells [54]. Moreover, MK866 increased the expression of RXR*α* whose heterodimerization with PPAR*γ* is thought to be necessary for the proapoptotic effect of PPAR*γ*. When MK866 was combined with ciglitazone and the RXR agonist, 13-cis-retinoic acid, there was a superadditive growth inhibitory effect compared to each drug alone [54]. These results suggest that the induction of PPAR*γ* and RXR*α* by MK866 sensitizes tumor cells to apoptosis by PPAR*γ* ligands or retinoids. In leukemia, lymphoma, and myeloma cells, exposure to rosiglitazone, 15d-PGJ_2_, or CDDO in combination with the RXR agonist, LG100268, or the retinoic acid receptor (RAR) agonist, all transretinoic acid, augmented the growth-inhibitory effects in these cells [55]. In agreement, treatment of breast cancer cells with another RXR selective ligand, AGN194204, and the PPAR*γ* ligand *γ*-linolenic acid showed an additive growth inhibitory response [65]. Thus, combining retinoids with PPAR ligands may prove to be a successful treatment in some cancers. This approach may be useful in the clinic as lower doses of each drug can be used to inhibit growth and a more optimal therapeutic index can be achieved.

Other drug combinations that show synergy with PPAR*γ* ligands include the platinum-based drugs. The combination of rosiglitazone with carboplatin in lung, ovarian, and colon cancer models showed a synergistic inhibition of growth. These results are PPAR*γ*-dependent as a non-TZD PPAR*γ* ligand was also able to enhance growth inhibition when combined with carboplatin, and the PPAR*γ* antagonist GW9662 significantly reduced the synergistic effect of rosiglitazone and carboplatin [56]. This synergy is related to the metallothioneins which are heavy metal binding proteins that play a role in platinum drug resistance. Rosiglitazone reduces metallothionein gene expression through a PPAR*γ*-dependent mechanism as treatment with the PPAR*γ* antagonist GW9662 abrogated metallothionein reduction by rosiglitazone and the non-TZD PPAR*γ* agonist GW1929 was also able to reduce metallothionein expression. Moreover, overexpression of the metallothionein MT1H reduced the synergistic effect of rosiglitazone and carboplatin. Therefore, it appears that the downregulation of metallothioneins contributes to the synergism of PPAR*γ* ligands and carboplatin. This synergistic effect of the combination of rosiglitazone and carboplatin was also observed in vivo in xenograft models of lung and ovarian cancer as well as a carcinogen-induced model of colon cancer [56]. Platinum-based drugs are currently being used in the clinic to treat lung and ovarian cancer, therefore, the use of PPAR*γ* ligands to enhance the efficacy of platinum drugs in the treatment of these cancers would be a great advancement in treating these two deadly diseases.

A dual PPAR*α*/*γ* ligand, TZD18, can induce apoptosis in adult lymphocytic leukemia and chronic myeloid leukemia cell lines [57, 58]. When TZD18 was combined with the bcr-abl tyrosine kinase inhibitor, imatinib, in these cell lines, there was enhanced growth inhibition. Treatment of leukemia patients with imatinib has been a successful therapy, however resistance to imatinib is a problem. These data suggest that combining TZD18 with imatinib is a potential therapy for treating imatinib resistant disease. In these studies, the growth inhibitory effects of TZD18 appeared to be independent of PPAR*α* or PPAR*γ* [57, 58].

A common mutation found in anaplastic thyroid cancer is a PAX8/PPAR*γ* rearrangement which results in downregulation of PPAR*γ*, suggesting that PPAR*γ* may be a tumor suppressor gene in this type of cancer [59]. In addition, the fusion protein, PAX8-PPAR*γ*, resulting from this rearrangement can act as a dominant negative inhibitor of wild-type PPAR*γ* [66]. Treatment with the novel PPAR*γ* agonist RS5444 in anaplastic thyroid cancer cells lines resulted in growth inhibition and PPAR*γ* activation. When RS5444 was combined with paclitaxel, a standard treatment for anaplastic thyroid cancer, enhanced apoptosis-inducing effects were observed [59]. Similarly, the combination of a PPAR*γ* agonist and docetaxel also exerted enhanced apoptosis-inducing and antitumor effects in human lung cancer cells. In addition, 15d-PGJ_2_ combined with docetaxel significantly reduced tumor volume compared with control, 15d-PGJ_2_, or docetaxel alone in both A549 and H460 xenografts. This combination showed a significant increase in apoptosis associated with inhibition of Bcl-2 and cyclin D1 expression and overexpression of caspase-3 and p53 pathway genes. However, enhanced expression of caspase 3 and inhibition of cyclin D1 by the combination was not reversed by GW9662, thus suggesting a possible PPAR*γ*-independent mechanism underlying enhanced apoptosis-inducing and antitumor effects by the combination of 15d-PGJ_2_ and docetaxel [60].

## 5. PPAR*γ* ANTAGONISTS EXERT APOPTOSIS-INDUCING EFFECTS

Although most of the antitumor effects of PPAR*γ* ligands are attributed to PPAR*γ* agonists, there is evidence that PPAR*γ* antagonists can have antiproliferative and apoptotic effects on tumor cells. In one study, two PPAR*γ* antagonists, T0070907 and GW9662, were tested in a panel of cancer cell lines and were able to inhibit cell growth and induce apoptosis. Combining the PPAR*γ* agonist, pioglitazone, with the PPAR*γ* antagonists T0070907 or GW9662 actually increased growth inhibition in a colon cancer cell line compared to each agent alone [67]. In breast cancer cells, the PPAR*γ* antagonist GW9662 inhibited growth and also surprisingly enhanced rosiglitazone-induced growth inhibition [68]. The effects of this enhanced growth inhibition appeared to be independent of PPAR*γ* activity as the combination of GW9662 and rosiglitazone did not result in activation of PPAR*γ* as compared to rosiglitazone alone which did activate PPAR*γ* [68]. How the combination of PPAR*γ* agonists and antagonists can enhance tumor growth inhibition needs further investigation.

Human primary squamous cellcarcinoma, lymph node metastasis, and squamous cell carcinoma cell lines express high levels of PPAR*γ* [69]. The specific PPAR*γ* antagonists T0070907, GW9662, and BADGE, but not agonists (i.e., pioglitazone and rosiglitazone) induced apoptosis in squamous cell carcinoma cell lines by interfering with adhesion to the extracellular matrix and disrupting survival signals, and thus inducing anoikis. Furthermore, the PPAR*γ* antagonists strongly inhibited the invasion of squamous cell carcinomas.These results imply a potentially important and novel role for the inhibition of PPAR*γ* function via the use of specific antagonists in the treatment of oral squamous cell carcinoma and the prevention of tumor invasion and metastasis [69]. Similarly, these antagonists also induced apoptosis in colorectal cancer cells as well as altered cell morphology which was linked to alterations in microtubules. The PPAR*γ* antagonists reduced the levels of *α* and *β* tubulin which prevented microtubule formation. This mechanism is unique from the known antimicrotubule drugs for the treatment of cancer such as the taxanes which alter microtubule polymerization. These data suggest that PPAR*γ* antagonists may be used as cancer therapy particularly in cancers that are not responsive to antimicrotubule therapy [70].

In contradiction to data suggesting that activation of PPAR*γ* can reduce tumor growth, treatment with PPAR*γ* ligands increased the number of colon tumors in the *Min* mouse model of familial adenomatous polyposis [71, 72]. In this model, PPAR*γ* may be playing a role in tumor promotion. Thus, it appears that activation or inhibition of PPAR*γ* can have dual roles in tumorigenesis depending on the type of cancer models examined. Determining the mechanisms of PPAR*γ* ligands in cancer either dependent or independent of PPAR*γ* action will be critical to understanding how to best target tumor cells for effective therapy.

## 6. ACTIVATION OF PPAR*γ* AS A MECHANISM FOR CERTAIN ANTICANCER AGENTS TO INDUCE APOPTOSIS

In addition to PPAR**γ** ligands, certain antitumor agents induce apoptosis through activation of endogenous PPAR*γ*. It was reported that the NSAID, sulindac, induced apoptosis, and upregulated PPAR*γ* expression in oral squamous carcinoma cells [73]. When PPAR*γ* was silenced with PPAR*γ* antisense oligonucleotides, sulindac lost its growth-inhibitory effects compared to control cells transfected with PPAR*γ* sense oligonucleotides in which significant growth inhibition was observed. Therefore, PPAR*γ* is an important mediator of cell growth induced by sulindac [73]. Similarly, *β*-carotene was shown to induce apoptosis and increase PPAR*γ* expression at both mRNA and protein levels in MCF-7 breast cancer cells. The presence of the PPAR*γ* antagonist GW9662 partially attenuated *β*-carotene-induced cell death, thus suggesting that PPAR*γ* is involved in *β*-carotene-induced apoptosis in this cell line [74].

Butyrate is a histone deacetylase inhibitor with the capacity to induce apoptosis of cancer cells. Its growth-inhibitory effects were suggested previously to be dependent on PPAR*γ* activation [75]. A recent study has shown that stimulation of cells with butyrate increased PPAR*γ* expression and activity as well as phospho-p38 MAPK protein levels and caspase-3 activity. Butyrate-induced upregulation of PPAR*γ* was abrogated by coincubation with the p38 MAPK inhibitor SB203580. Treatment of cells with butyrate resulted in both increased caspase-8 and -9 activity and reduced expression of XIAP and survivin. Moreover, these effects were almost completely abolished in cells expressing a dominant-negative PPAR*γ* mutant [76]. These results collectively suggest PPAR*γ* as a key target in the butyrate-induced signaling cascade leading to apoptosis.

Capsaicin (N-vanillyl-8-methyl-alpha-nonenamide), a spicy component of hot pepper, is a homovanillic acid derivative that preferentially induces certain cancer cells to undergo apoptosis and has a putative role in cancer chemoprevention. In colon cancer cells, capsaicin induced apoptotic cell death; this effect was completely blocked by bisphenol A diglycidyl ether, a specific PPAR*γ* antagonist, but not by capsazepine, a specific antagonist for vanilloid receptor [77]. Thus, it seems that capsaicin-induced apoptotic cell death in colon cancer cells is associated with the PPAR*γ* pathway without the involvement of the vanilloid receptor.

Abnormally elevated expression or activation of cyclooxygenase-2 (COX-2) is often associated with cell proliferation and transformation. However, increased numbers of studies have suggested that induction of COX-2 can be proapoptotic [78–81]. In COX-2-mediated apoptosis, production of prostaglandin D_2_(PGD_2_) and 15d-PGJ_2_ and activation of PPAR*γ* have been considered important mechanisms. For example, the alkylphospholipid type antitumor agent ET-18-O-CH3, at the same concentration ranges that induce apoptosis, induced COX-2 expression in H-ras transformed human breast epithelial cells (MCF10A-ras). The addition of a selective COX-2 inhibitor SC-58635 and COX-2 gene knockdown blocked ET-18-O-CH3-induced apoptosis, suggesting that COX-2 induction by this drug is causally linked to its apoptosis-inducing activity. ET-18-O-CH3 treatment resulted in elevated release of 15d-PGJ_2_ and DNA binding and transcriptional activity of PPAR*γ*. These data suggest that ET-18-O-CH3 likely induces COX-2 expression and production of 15d-PGJ2, leading to induction of apoptosis in MCF10A-ras cells [79].

In agreement, several chemotherapeutics including paclitaxel, cisplatin, and 5-fluorouracil induced COX-2 expression and prostaglandin (PG) synthesis, accompanied by a substantial decrease of viability and enhanced apoptosis [80]. Cells were significantly less sensitive to apoptotic death when either COX-2 expression or its activity was suppressed by siRNA or by the selective COX-2 inhibitor NS-398. Experiments performed to clarify how COX-2 leads to apoptosis revealed a profound proapoptotic action of PGD_2_ and its dehydration product, 15d-PGJ_2_, because chemotherapeutic-induced apoptosis was prevented by siRNA targeting lipocalin-type PGD synthase (L-PGDS), which catalyzes the isomerization of PGH_2_ to PGD_2_. Moreover, apoptosis by chemotherapeutics, PGD_2_ and 15d-PGJ_2_, was suppressed by the PPAR*γ* antagonist, GW-9662 or PPAR*γ* siRNA. Collectively, this study suggests that COX-2 induction and synthesis of L-PGDS-derived PPAR*γ*-activating PGs are a decisive target by which several chemotherapeutics induce apoptosis [80]. As well, the novel natural compound, a cycloanthranilylproline derivative (Fuligocandin B) was recently reported to sensitize leukemia cells to TRAIL-induced apoptosis through COX-2-dependent 15d-PGJ_2_ production. However, the synergy mediated by 15d-PGJ_2_ works in a PPAR*γ*-independent manner as PPAR*γ* siRNA failed to block the synergy [82].

These findings have important clinical impact on the treatment of cancer patients if this mechanism, particularly to chemotherapeutic agents, is common in different types of cancer cells. Because NSAIDs possess COX-2-inhibitory activity and are commonly used by many people including cancer patients, caution should be taken during cancer chemotherapy to avoid potentially diminished therapeutic efficacy due to COX-2 inhibition.

## 7. CONCLUSIONS

The role of PPARs, particularly PPAR*γ*, in cancer is an evolving field. Understanding of the molecular mechanisms underlying PPAR-mediated regulation of apoptosis of tumor cells will continue to expand. Accordingly, targeting PPARs, especially PPAR*γ* for cancer chemoprevention and therapy may prove to be very effective and will remain an interesting research topic. As we continue to address specific signaling pathways that lead to cancer, we can further elucidate how PPARs and their ligands contribute to these pathways and design effective combinations of therapy that target multiple steps in the oncogenic process. PPAR*γ* ligands have the potential to sensitize cancer cells to or overcome resistance to chemotherapy or other anticancer drug-based therapies. Thus, exploring mechanism-driven PPAR*γ* ligand-based combination regimens for both cancer chemoprevention and therapy should be the focus of this future study. Specific types of tumors and unique tumor microenvironments also behave differently to PPAR activation or inhibition. Therefore, a close examination of individual tumor types and their response to PPAR stimulation will be critical for successful cancer therapy targeting PPARs, particularly PPAR*γ*. Many studies have revealed that TZDs exert PPAR*γ*-independent effects on induction of apoptosis in various cancer cells. Although some of the TZDs are clinically used drugs for treatment of diabetes with acceptable or manageable side effects or toxicity, they were not originally developed as anticancer drugs and hence are not optimal for cancer treatment. Therefore, it is necessary to use them as lead compounds for synthesizing analogs as anticancer drugs that possess better or optimized cancer chemopreventive or therapeutic efficacy.

## Figures and Tables

**Table 1:** PPAR*γ* agonists induce apoptosis in cancer.

PPAR*γ* agonist	PPAR*γ*	Tumor type	Molecular mediator(s) of apoptosis	Reference
15d-PGJ_2_	Independent	Breast	Unknown	[8]
Troglitazone and 15d-PGJ_2_	Dependent	Thyroid	*c-myc*	[9]
Ciglitazone	Dependent	Thyroid	PPAR*γ*	[10]
Rosiglitazone	Dependent	Thyroid	NF-*κ*B, cyclinD1, caspase-3	[11]
Troglitazone and 15d-PGJ_2_	Unknown	Colon	*c-myc, c-jun,* GADD153	[12]
Troglitazone	Dependent	Lung	GADD153	[13]
Troglitazone	Independent	Colon	EGR-1, NAG-1	[14, 15]
15d-PGJ_2_	Dependent	Colon	EGR-1, NAG-1	[15]
Troglitazone	Dependent	Lung	ERK1/2	[16]
Troglitazone	Dependent and independent	Colon	p53, POX	[17]
Troglitazone	Independent	Prostate	Bcl-2, Bcl-X_*L*_	[18]
15d-PGJ_2_	Independent	Oral	Stat3	[19]
15d-PGJ_2_	Independent	Prostate, bladder	Caspase-3, -7	[20]
15d-PGJ_2_	Independent	Multiple myeloma, burkitt lymphoma	NF-kappa-B, cIAP-1, XIAP, c-FLIP	[21]
Rosiglitazone	Dependent	Breast	PPAR*γ*, p53	[22]

**Table 2:** Combination of anticancer drugs with PPAR*γ* ligands enhances tumor cell death.

PPAR*γ* agonist + antitumor agent	PPAR*γ*	Tumor type	Molecular mediator(s) of apoptosis	Reference
Troglitazone or ciglitazone or GW1929 + TRAIL	Independent	Lung	DR5, c-FLIP	[34]
15d-PGJ_2_ + TRAIL	Independent	Leukemia, prostate	DR5	[47]
Troglitazone + TRAIL	Independent	Glioblastoma, neuroblastoma	c-FLIP, survivin, DR5	[52]
Rosiglitazone + TRAIL	Independent	Renal, glioma, breast, prostate	ROS, DR5, c-FLIP	[49]
15d-PGJ_2_ or ciglitazone or troglitazone or CDDO or CDDO-Me + TRAIL	Independent	Prostate, ovarian, colon	c-FLIP	[50]
Troglitazone + TRAIL or troglitazone + etoposide or paclitaxel	Independent	Glioma	PTP1B, STAT3, c-FLIP, Bcl-2	[53]
15d-PGJ_2_ + MK886	Dependent	Lung	PPAR*γ*, RXR*α*	[54]
15d-PGJ_2_ + Indomethacin				
Ciglitazone + MK886 + 13-cis-retinoic acid				
Rosiglitazone + LG100268 or all transretinoic acid	Dependent and independent	Leukemia, lymphoma, myeloma	Bcl-2, caspase-9	[55]
15d-PGJ_2_ + LG100268 or all trans-retinoic acid				
CDDO + LG100268 or all transretinoic acid				
Rosiglitazone + carboplatin	Dependent	Lung, ovarian, colon	MT1H, MT1X, MTIIA	[56]
TZD18 + imatinib	Independent	Leukemia	Bax, NF-*κ*B	[57, 58]
RS5444 + paclitaxel	Dependent	Thyroid	p21WAF1/CIP1	[59]
15dPGJ_2_ + docetaxel	Independent	Lung	Bcl-2, BAD, cyclin D1, p53	[60]
